# Effect of pioglitazone in acute ischemic stroke patients with diabetes mellitus: a nested case–control study

**DOI:** 10.1186/s12933-019-0874-5

**Published:** 2019-05-31

**Authors:** Min-Hee Woo, Hye Sun Lee, Jinkwon Kim

**Affiliations:** 1 Department of Neurology, CHA Bundang Medical Center, CHA University College of Medicine, Seongnam, Republic of Korea; 2 Biostatistics Collaboration Unit, Yonsei University College of Medicine, Seoul, Republic of Korea; 3 Departments of Neurology, Gangnam Severance Hospital, Yonsei University College of Medicine, 211 Eonjuro, Gangnam-gu, Seoul, 06273 Republic of Korea

**Keywords:** Ischemic stroke, Diabetes mellitus, Secondary prevention, Pioglitazone

## Abstract

**Background:**

Pioglitazone is an oral antidiabetic drug with multiple pleiotropic actions. Recent clinical trials have demonstrated that treatment with pioglitazone reduces cardiovascular risk in patients who have had an ischemic stroke. We examined the secondary preventive effects of pioglitazone in acute ischemic stroke patients with diabetes mellitus (DM) based on nationwide real-world data.

**Methods:**

A nested case–control study was conducted with data from the National Health Insurance Service-National Sample Cohort in Korea. Study subjects were diabetic patients admitted for acute ischemic stroke (ICD-10 code; I63) between 2002 and 2013. Cases were defined as patients who suffered from composites of recurrent stroke (I60–63), myocardial infarction (I21), or all-cause mortality after ischemic stroke. Controls were selected by incidence density sampling. Three controls were matched to each case for sex, age, treatment with insulin, and oral antidiabetic medications, with the exception of pioglitazone. Medication history after ischemic stroke was obtained by accessing the prescription records. In the matched dataset, conditional logistic regression analysis was performed with adjustments for hypertension, atrial fibrillation, prior myocardial infarction, and treatment with oral antithrombotics and statins.

**Results:**

From the patients with acute ischemic stroke and DM, 1150 cases with primary outcomes were matched to 3450 controls. In the matched analysis, treatment with pioglitazone was significantly associated with a lower cardiovascular risk (adjusted OR [95% CI], 0.43 [0.23–0.83]).

**Conclusions:**

In this nested case–control study using real-world data, treatment with pioglitazone exhibited significant cardiovascular preventive effect in diabetic patients with acute ischemic stroke.

## Background

Stroke is the major cause of death and long-term disability worldwide [1]. Even with advances in modern medicine, stroke survivors have a greater risk of recurrent stroke and cardiovascular disease [2]. Recurrent events are more likely to be more disabling or fatal than the first event [3]. Diabetes mellitus (DM) is a common and well-established risk factor for stroke. Stroke patients with DM have a poor short- and long-term prognosis compared with non-diabetic stroke patients [4]. For diabetic stroke patients at high risk, effective cardiovascular preventive strategies remain a major clinical challenge. Pioglitazone is an oral antidiabetic medication belonging to the drug class known as thiazolidinediones, and it acts as an agonist of the peroxisome proliferator-activated receptor γ (PPARγ). In addition to lowering serum glucose, pioglitazone has several pleiotropic effects, including reducing insulin resistance, inducing vascular inflammation, and improving endothelial dysfunction and dyslipidemia, which may lead to cardiovascular protection [5]. Two large randomized trials have suggested that treatment with pioglitazone reduces cardiovascular risk in patients with DM or insulin resistance who had suffered from an ischemic stroke or a transient ischemic attack (TIA) [6, 7]. In real-world data, the impact of pioglitazone may vary depending on different clinical characteristics of patients and interaction with other antidiabetic medications. Furthermore, the cardiovascular preventive effect of pioglitazone in Asian stroke survivors are not well known [8]. The aim of this study is to evaluate the cardiovascular effect of pioglitazone in acute ischemic stroke patients with DM using nationwide health claims data in Korea.

## Methods

### Study design and data source

We conducted a nested case–control study using the National Health Insurance Service-National Sample Cohort (NHIS-NSC) in Korea [9]. All Koreans are eligible for and required to have universal health coverage under the NHIS. The NHIS-NSC consisted of 1,025,340 participants who were selected from the NHIS database in 2002 using a stratified random sampling method based on sex, age, and household income level (2.2% of the total eligible Korean population in 2002). The NHIS-NSC database contains health insurance claims for hospital visits, medical procedures, prescription records (name, dose, duration, and date), clinical diagnoses, and clinical characteristics including sex, age, household income, and death statistics. Diagnoses from each hospital visit were recorded according to the International Statistical Classification of Diseases, 10th revision (ICD-10). The NHIS-NSC data were fully anonymized in order to protect privacy. Due to the nature of a retrospective study with anonymized data, this study was approved and the requirement for informed consent was waived by the Institutional Review Board of Bundang CHA Medical Center (CHAMC 2018-12-032).

### Study subjects

We established a cohort of patients aged ≥ 20 years old with DM and acute ischemic stroke, defined as hospitalization (admission or emergency department visit) with a primary diagnosis of ischemic stroke (I63) between 2002 and 2013. We only included patients who had undergone brain CT or MRI during hospitalization because of the assumption that those with acute ischemic stroke should undergo brain imaging [10]. The index date of the cohort was the starting date of hospitalization. The presence of DM is defined as the presence of ICD-10 codes (E08–11, E13–14) and at least one prescription of antidiabetic medication with diagnosis before or upon hospitalization. Based on the health claims data of the NHIS-NSC, enrolled patients were followed up until the development of a primary outcome, the loss of eligibility for NHIS, death, or the study end date (Dec 31, 2013). Patients followed up for < 1 month were excluded. To avoid the confounding effect of rosiglitazone, another drug of the thiazolidinedione class withdrawn from the Korea’s market in 2010 due to concerns of its increased cardiovascular risk, we excluded patients who had been exposed to rosiglitazone for ≥ 1 month during the study period.

### Clinical outcomes

The primary outcome was a composite of recurrent stroke, myocardial infarction (MI), and all-cause death, whichever one occurred earliest. Patients were diagnosed with recurrent stroke if they were re-hospitalized with a primary diagnosis of I60–63 and underwent a brain CT or an MRI during their hospital visit. MI was defined as hospitalization with a primary diagnosis of I21. The diagnostic accuracy of I60–I63 for stroke in the NHIS has been reported to be over 80% in prior validation studies [11, 12]. The accuracy of I21 for MI in the NHIS has been reported to be 73–93% [13, 14]. Death statistics in stroke patients are considered to be reliable [15].

### Selection of cases and controls

To construct a nested case–control study, we defined cases as patients who suffered from the primary outcome during the follow-up period. For each case, we sampled 3 controls with replacement from the cohort, who were required to be event-free and at risk on the date of the primary outcome of their matched case, by incidence density sampling. Controls were required to be of the same sex and age as their matched case and to be taking the same medications at the time of case occurrence, that is, insulin and oral antidiabetic drugs, with the exception of pioglitazone.

### Covariates

For baseline characteristics, we collected data on sex, age, and risk factors at index stroke. The presence of hypertension (I10–15), atrial fibrillation (I48), and prior MI (I21) was defined by the diagnostic code of ICD-10 in the NHIS-NSC before or at hospitalization of index stroke. Hypertension was considered relevant only if the subject was prescribed antihypertensive drugs (calcium-channel blockers, angiotensin-converting enzyme inhibitors, angiotensin-receptor blockers, diuretics, and beta-blockers) with the diagnostic code [45].

### Assessment of medications

We collected prescription records of pioglitazone, other oral antidiabetic medications (sulfonylurea, biguanide, dipeptidyl peptidase-4 inhibitor, and alpha-glucosidase inhibitor), and insulin during the study period. Treatment with oral antithrombotics (antiplatelet or anticoagulant: aspirin, clopidogrel, ticlopidine, triflusal, cilostazol, warfarin, rivaroxaban, apixaban, and dabigatran) and statins (atorvastatin, fluvastatin, lovastatin, pitavastatin, pravastatin, rosuvastatin, and simvastatin) was also evaluated based on prescription records. Sodium-glucose co-transporter-2 inhibitors and edoxaban were not available in Korea during the study period. At times of primary outcomes in cases and matched controls, treatment with these oral medications was determined by exposure within the past 30 days. Because the duration of treatment with insulin could not be directly obtained from prescription records in the NHIS-NSC (example of insulin prescription record: [regular insulin, 100 U/mL, 10 mL Vial] × 2 on May 9, 2011), unlike oral medications, treatment with insulin was determined by the presence of insulin prescriptions within the past 90 days.

### Statistical analyses

To estimate the odds ratio (OR) for primary outcomes with pioglitazone treatment, we performed conditional logistic regression with the matched case–control groups. As covariates, the presence of hypertension, atrial fibrillation, prior MI, treatment with oral antithrombotics, and statins were adjusted in the multivariate model. Data management and statistical analyses were performed with the SAS statistical software package, version 9.4 (SAS Institute Inc., Cary, NC, USA) and R software, version 3.4.3 (The R Foundation for Statistical Computing, Vienna, Austria; http://www.R-project.org/). A two-sided p value of < 0.05 was considered statistically significant.

## Results

**Fig. 1:**
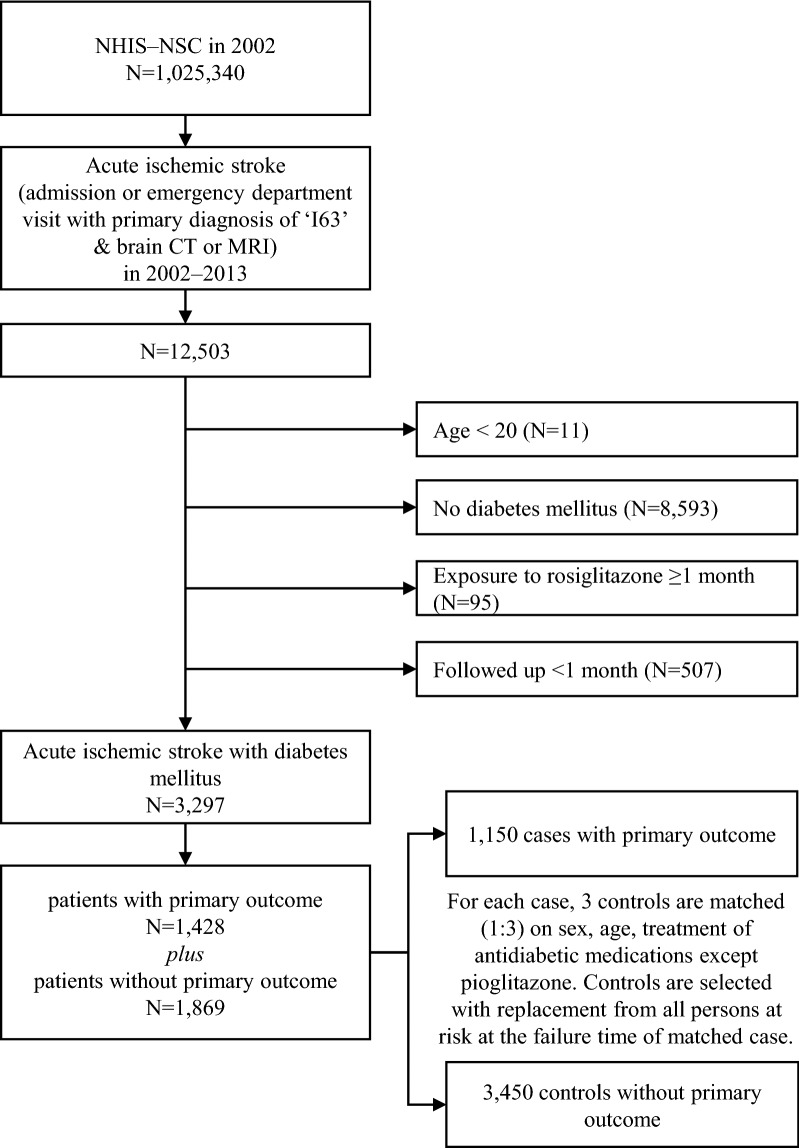
Flow diagram for the selection of cases and controls in a nested case–control study design. *NHIS*-*NSC* the National Health Insurance Service-National Sample Cohort in Korea

In the NHIS-NSC, there were 12,503 patients with acute ischemic stroke (Fig. 1). In accordance with the inclusion and exclusion criteria, we constructed a cohort of 3297 patients with acute ischemic stroke and DM. The mean post-stroke follow-up period of patients was 3.1 ± 2.5 years (mean ± standard deviation). During the post-stroke follow-up period, there were 1428 patients who suffered from a primary outcome. To construct a nested case–control study, we randomly selected 3 controls with replacement who were event-free and matched each one with a case having a primary outcome. After the matching process, we finally included 1150 patients with primary outcomes as cases and 3450 patients without primary outcomes as controls. Figure 1 displays the flow diagram for the case and control selection process with a nested case–control design.

**Fig. 2:**
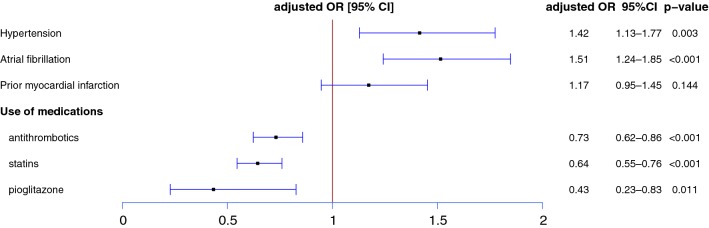
Risk factors for primary outcomes in the patients with acute ischemic stroke and diabetes mellitus. Primary outcome is defined as composites of recurrent stroke, myocardial infarction, or all-cause death after acute ischemic stroke. Cases and controls are matched for same sex, age, and treatment with sulfonylurea, biguanide, dipeptidyl peptidase 4 inhibitor, alpha-glucosidase inhibitor, and insulin. Adjusted OR (odds ratio), 95% CI (confidence interval) and p value are derived from conditional logistic regression analyses, which included the listed variables

Clinical characteristics of the selected cases and controls are shown in Table 1. Due to the matching process, there were no differences between the cases and the controls in sex, age, and treatment with antidiabetic medications (sulfonylurea, biguanide, dipeptidyl peptidase-4 inhibitor, alpha-glucosidase inhibitor, and insulin) except pioglitazone. Treatment with pioglitazone was more frequent in cases compared to controls (2.1% vs 1.0%). When we performed conditional logistic regression adjusted for hypertension, atrial fibrillation, prior MI, and treatment with oral antithrombotics and statins (Fig. 2), we found that treatment with pioglitazone was significantly associated with a lower risk of having a primary outcome (adjusted OR 0.43, 95% CI [0.23–0.83], p = 0.011). Along with pioglitazone, treatment with antithrombotics (adjusted OR 0.73, 95% CI [0.62–0.86]) and statins (adjusted OR 0.64, 95% CI [0.55–0.76]) showed significant cardiovascular preventive effects in acute ischemic stroke patients with DM.

**Table 1:** Characteristics of cases and matched controls

Variables	Cases (N = 1150)	Controls (N = 3450)	Crude OR [95% CI]	p
Sex, male	578 (50.3)	1734 (50.3)	–	
Age	70–74 [65–69; 75–79]	70–74 [65–69; 75–79]	–	
Hypertension	1039 (90.3)	3005 (87.1)	1.40 [1.12–1.75]	0.003
Atrial fibrillation	184 (16.0)	398 (11.5)	1.49 [1.22–1.80]	< 0.001
Prior myocardial infarction	140 (12.2)	358 (10.4)	1.20 [0.97–1.48]	0.088
Use of medications				
Antithrombotics^a^	713 (62.0)	2391 (69.3)	0.68 [0.58–0.79]	< 0.001
Statins	276 (24.0)	1156 (33.5)	0.61 [0.52–0.71]	< 0.001
Sulfonylurea	411 (35.7)	1233 (35.7)	–	
Biguanide	379 (33.0)	1137 (33.0)	–	
Dipeptidyl peptidase 4 inhibitor	34 (3.0)	102 (3.0)	–	
Alpha-glucosidase inhibitor	86 (7.5)	258 (7.5)		
Pioglitazone	11 (1.0)	74 (2.1)	0.44 [0.23–0.83]	0.012
Insulin	347 (30.2)	1041 (30.2)	–	

Cases and controls (1:3) are matched for same sex, age, and treatment with sulfonylurea, biguanide, dipeptidyl peptidase 4 inhibitor, alpha-glucosidase inhibitor, and insulin

Crude OR (odds ratio), 95% CI (confidence interval) and p values are derived from conditional logistic regression analyses

^a^Antithrombotics include aspirin, clopidogrel, ticlopidine, triflusal, cilostazol, warfarin, rivaroxaban, apixaban, and dabigatran

### Secondary analysis for individual outcome

Among the 1150 cases with primary outcomes, the number of patients with recurrent stroke, MI and all-cause death was 428, 50, and 672, respectively. We performed a conditional logistic regression analysis for the three subgroups consisting of cases with the individual outcome and matched controls (Table 2). In the secondary analysis, treatment with pioglitazone was significantly associated with reduced risk for all-cause death.

**Table 2:** Secondary analysis for individual outcome according to treatment with pioglitazone

Outcomes	Number of cases	Adjusted OR [95% CI]
Recurrent stroke	428	0.70 [0.31–1.61]
Myocardial infarction	50	NA^a^
All-cause death	672	0.27 [0.09–0.79]

For each case, three controls are matched for same sex, age, and treatment with sulfonylurea, biguanide, dipeptidyl peptidase 4 inhibitor, alpha-glucosidase inhibitor, and insulin. Adjusted OR (odds ratio) and 95% CI (confidence interval) for ‘treatment with pioglitazone’ are derived from conditional logistic regression analyses with adjustments for hypertension, atrial fibrillation, prior myocardial infarction, treatment with antithrombotics and statins

^a^Not applicable due to no case of myocardial infarction in the arm of ‘treatment with pioglitazone’

### Analysis considering burden of antidiabetic medications

In this nested case–control design matched for oral antidiabetic medications other than pioglitazone, there is a potential concern that the beneficial effect of pioglitazone may reflect more aggressive treatment with combinations of antidiabetic treatments, rather than the class effect of pioglitazone (the difference in risk between biguanide and biguanide plus pioglitazone may be due to the higher medication burden, rather than pioglitazone itself). To evaluate this possibility, we reconstructed a new nested case–control model in which each case was matched with 3 controls for sex, age, treatment with insulin, and the number of oral antidiabetic medications taken, including pioglitazone. In this model, cases and matched controls were prescribed the same number of oral antidiabetic medications (a patient taking biguanide plus pioglitazone could be matched with a patient taking biguanide plus sulfonylurea or any other combination of two classes of oral antidiabetic drugs). The median number of oral antidiabetic medications taken in both cases and controls was 1 [0–2] (median [interquartile range]). In the model in which cases were matched for the number of oral antidiabetic medications taken, the preventive effect of pioglitazone was also significant (adjusted OR 0.40, 95% CI [0.20–0.83], p = 0.013).

### Event-free survival curve

**Fig. 3:**
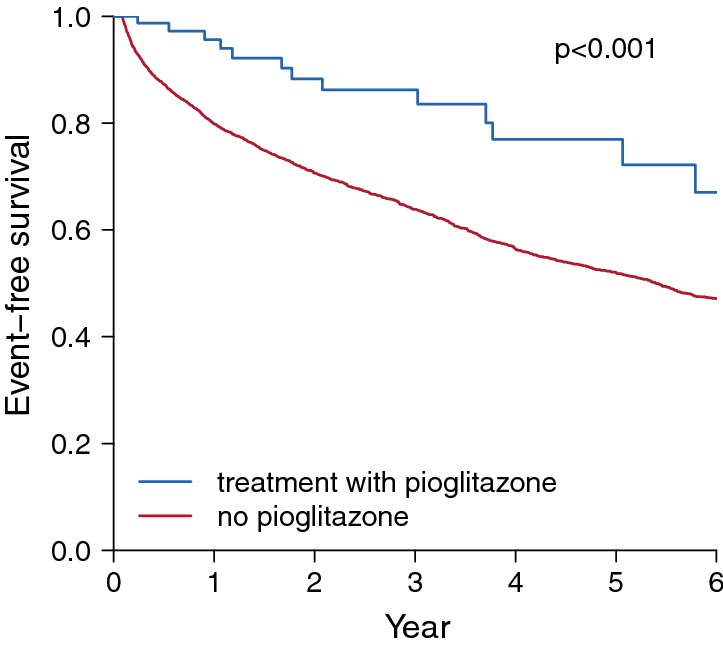
Event-free survival plot according to treatment with pioglitazone. The plot illustrates an estimated event-free survival curve according to treatment with pioglitazone (defined as exposure within the past 30 days) during the followed-up period after acute ischemic stroke. p value is obtained from the Mantel–Byar test which compares survival curves by treatment with pioglitazone

To help understand the effect of pioglitazone, we illustrate an event-free survival plot according to treatment with pioglitazone based on the full cohort of patients with acute ischemic stroke and DM (N = 3297, Fig. 1). Accounting the nature of time-dependent variable of treatment with pioglitazone during follow-up period in the cohort, estimated event-free survival is visualized using the method of Simon and Makuch [16, 17]. When we compared the survival curves by the Mantel–Byar test, there is a significantly reduced risk for the primary outcome with pioglitazone (Fig. 3) [18].

## Discussion

In this nested case–control study using nationwide health insurance claims data, treatment with pioglitazone was significantly associated with a lower risk of a primary outcome in diabetic patients with acute ischemic stroke. This finding is in line with prior studies reporting on the protective role of pioglitazone against cardiovascular disease. Treatment with pioglitazone, an antidiabetic medication currently available, may further reduce the residual cardiovascular risk in high-risk patients with prior stroke and DM. In addition, we demonstrated the applicability of pioglitazone’s preventive effect in treating Asian stroke patients.

### Prior clinical trials for cardiovascular effect of pioglitazone

There have been two large randomized clinical trials evaluating the cardiovascular preventive effects of pioglitazone. The PROspective pioglitAzone Clinical Trial In macroVascular Events (PROactive) study was a randomized trial which investigated the secondary preventive effect of pioglitazone in 5238 patients with type 2 diabetes and macrovascular disease [19]. Compared with our study, which included patients with acute ischemic stroke, the PROactive study included patients who had been more than 6 months since the onset of MI or stroke before study entry. Although the PROactive study failed to show a significant benefit of pioglitazone from primary outcome analysis, there was a significantly reduced rate of fatal or nonfatal stroke with pioglitazone treatment in a subgroup analysis of patients with prior stroke (hazard ratio (HR) 0.53, 95% CI 0.34–0.85, p = 0.009) [7]. In subgroups without prior stroke, there was no significant preventive effect of pioglitazone on the development of stroke (HR 1.06; 95% CI 0.73–1.52, p = 0.767). The Insulin Resistance Intervention After Stroke (IRIS) trial, performed more recently, was a randomized clinical trial based on the hypothesis that pioglitazone may reduce cardiovascular risk in non-diabetic patients with recent ischemic stroke or TIA (within 6 months) and insulin resistance [6]. It is well established that people with insulin resistance are at a higher risk of cardiovascular disease, even if non-diabetic, and insulin resistance is common in ischemic stroke patients [20]. Pioglitazone has been shown to be effective in improving both glycemic control and insulin-resistance [21]. The IRIS trial showed that treatment with pioglitazone can reduce the risk of both acute coronary events and ischemic stroke in insulin resistant patients who had a recent ischemic stroke or TIA [22, 23]. In this IRIS trial, primary outcomes of fatal or non-fatal stroke or myocardial infarction had occurred in 9.0% of the pioglitazone group and 11.8% of the placebo group by 4.8 years into the study period (HR 0.76, 95% CI 0.62–0.93, p = 0.007). The Juntendo Stroke Prevention study in Insulin Resistance and Impaired glucose Tolerance (J-SPIRIT) study was another randomized trial consisting of 120 patients with impaired glucose tolerance or newly-diagnosed DM in Japan who had experienced a non-disabling ischemic stroke or a TIA [24]. Over a median follow-up period of 2.8 years, treatment with pioglitazone was associated with a lower risk of recurrent stroke (HR 0.62, 95% CI 0.13–2.25, p = 0.49), although the difference was not statistically significant and the trial was underpowered to evaluate the effect of pioglitazone. A meta-analysis of the above three trials demonstrated that treatment with pioglitazone in stroke patients with insulin resistance, prediabetes, and DM was associated with a significantly lower risk of recurrent stroke (HR 0.68, 95% CI 0.50–0.92, p = 0.01) and major vascular events (HR 0.75, 95% CI 0.64–0.87, p < 0.01) [25].

### Meta-analysis and observational studies for cardiovascular effect of pioglitazone

Similarly, a meta-analysis of ten randomized controlled trials with patients with cardiovascular disease found a significantly reduced risk for recurrent major adverse cardiovascular events with pioglitazone treatment (relative risk 0.74, 95% CI 0.60–0.92) [26]. Another meta-analysis of nine randomized trials found that treatment with pioglitazone was associated with a lower risk of cardiovascular events in patients with pre-diabetes or insulin resistance (relative risk 0.77, 95% CI 0.64–0.93) and diabetes (relative risk 0.83, 95% CI 0.72–0.97) [27]. In addition to the data from these randomized trials, the preventive effect of pioglitazone on cardiovascular disease was also demonstrated in observational studies [28, 29]. From real-world data based on Taiwan’s National Health Insurance Research Database of type 2 DM patients newly prescribed oral antidiabetic medications, pioglitazone users were at a significantly lower risk of cardiovascular events than non-pioglitazone users (adjusted HR 0.61, 95% CI 0.50–0.75) [30].

### Magnitude of the cardiovascular risk reduction by pioglitazone

In line with previous reports, our findings provide real-world evidence of the preventive effect of pioglitazone on cardiovascular disease in patients with acute ischemic stroke and DM. Based on our data, the magnitude of the risk reduction by pioglitazone (adjusted OR 0.43) seemed to be greater than those determined in prior randomized trials (HR 0.53 in PROactive, HR 0.76 in IRIS). The more favorable effect observed in our study may originate from the differences in the clinical characteristics of the study population. Unlike randomized trials, real-world data included heterogenous patients with multiple comorbidities. Compared with the PROactive study (which included diabetic patients with myocardial infarction or stroke > 6 months before study entry) or the IRIS study (which included patients with ischemic stroke or TIA with insulin resistance), the diabetic patients hospitalized for acute ischemic stroke in this study may receive a greater benefit from pioglitazone with respect to their underlying higher risk [31]. While the main data reported in the PROactive and IRIS trials were based on the ‘intention-to-treat’ analysis regardless of the compliance to the treatment, current study evaluated the treatment effect of pioglitazone based on the actual prescription records during the study period. In the post hoc analysis of IRIS trial regarding adherence to pioglitazone (‘on-treatment analysis’), the risk reduction in those with good adherence was more greater (HR [95% CI] were 0.57 [0.39–0.84] for stroke/MI compared to placebo) than the estimation by the ‘intention-to-treat’ analysis [32].

### Cardiovascular protective mechanisms of pioglitazone

Currently, the exact mechanism of how pioglitazone reduces cardiovascular risk is still unclear. Pioglitazone is principally a PPARγ agonist and partially an activator of PPARα, whose actions improve glycemic control and insulin sensitivity. Pioglitazone, through its PPARγ agonist actions, regulates gene expression in multiple insulin-sensitive tissues, lipid-metabolism, and inflammation [33–36]. Independent of glycemic control, pioglitazone has multiple positive effects on fat distribution, coagulation, thrombosis, lipid and protein composition, and vascular inflammation [6]. Upon treatment with pioglitazone, overall favorable changes are noted, including decreases in blood pressure, triglycerides, and C-reactive protein and increases in insulin sensitivity, high-density lipoprotein-cholesterol, adiponectin, and apolipoprotein A–I, as well as regulation of the balance between regulatory and effector T cells [37–41]. Treatment with pioglitazone reduced the oxidation of high-density lipoproteins, but not low-density lipoprotein in patients with type 2 diabetic mellitus [42]. Pioglitazone may contribute to the attenuation and stabilization of atherosclerotic plaque inflammation, alteration of atherosclerotic core composition, reduction of the necrotic core, and prevention of intimal hyperplasia after coronary stent implantation [22, 27, 43–46]. Treatment with pioglitazone can slow the progression of carotid intima-media thickening and improve endothelial function [37, 47, 48]. The beneficial effects of pioglitazone are apparent regardless of the use of other antidiabetic medications or insulin [19]. Although we were unable to identify the underlying mechanism of action of pioglitazone, our finding suggests that improvement in insulin resistance and high pleiotropic potency of pioglitazone could contribute to reducing residual cardiovascular risk in diabetic patients with acute ischemic stroke. Further study is needed to elucidate even more of the promising effects of pioglitazone on prevention of cardiovascular disease.

### Strength and limitation

This study had strengths and limitations. By using nationwide health insurance claims data, we could evaluate the long-term, real-world prognosis of acute ischemic stroke patients with DM according to the treatment with pioglitazone. Unlike well-designed clinical trials, changes in drug medications and non-adherence to medications are common in clinical practice. Because the NHIS in Korea is a single payer insurer, we were able to access detailed prescription data for treatment with pioglitazone and other antidiabetic medications throughout the post-stroke period. For proper control of serum glucose, DM patients frequently need to receive multiple classes of antidiabetic medications. Concomitant use of other classes of antidiabetic medications could act as a barrier to evaluate the effect of the individual medications. To minimalize the influence of concomitant antidiabetic medications, we conducted a nested case–control study which matched cases and controls according to their use of other antidiabetic medications. The significant reduction of primary outcomes observed in this matched dataset points to the independent benefit of pioglitazone for secondary prevention, regardless of concomitant antidiabetic medications. However, with the limitation of a retrospective study design, we should also consider the possibility of hidden bias between patients who received pioglitazone and those who did not. In this study, only a small portion of patients received pioglitazone, which could result in biased estimates. The low usage of pioglitazone may reflect the non-adherence to antidiabetic medications and the functional deficit of stroke patients. Nevertheless, the large portion of non-pioglitazone-treated patients could be potential targets for effective risk reduction by treatment with pioglitazone.

Because the current study constructed matched dataset for treatment with antidiabetic medications, we could not evaluate the potential effect of individual class except pioglitazone and various combinations of antidiabetic medications. Besides pioglitazone, other class of antidiabetic medications may provide various cardiovascular effects, not merely on the development of acute cardiovascular events. A nationwide cohort study from the Taiwan National Health Insurance Database demonstrated that combinations of thiazolidinediones plus metformin and alpha-glucosidase inhibitors plus metformin were associated with a lower risk of major adverse cardiovascular event when compared with sulfonylurea plus metformin [49]. Another real world data from the Taiwan National Health Insurance Database showed that treatment with dipeptidyl peptidase-4 inhibitor was associated with a lower risk of new-onset atrial fibrillation which is the most common cause of cardioembolic stroke [50]. In a network analysis from 11 randomized controlled trials, gliclazide (a type of sulfonylureas) was the only medication that significantly reduced left ventricular mass in type 2 diabetes [51]. Recent clinical trials have documented that treatment with glucagon-like peptide-1 receptor agonists, which is not available during the current study period in Korea, can lead to marked risk reduction in major adverse cardiovascular events [52]. Overall, it is necessary to conduct more research to evaluate cardiovascular effect and action mechanism of the antidiabetic agents.

This study was performed with Asian stroke patients with DM. The individual characteristics of stroke patients and their responses to medication may differ with race and ethnicity [53]. In addition, our study did not provide data on the effect of pioglitazone in patients without stroke. Therefore, our results should be interpreted with caution, and the general applicability of pioglitazone to different populations should be further evaluated. There were also limitations of study from health claims data. Due to the lack of detailed clinical information in health claims data, we could not collect data on the severity of index stroke, radiological findings, blood pressure, duration of diabetes mellitus, levels of glucose, Hemoglobin A1c and low-density lipoprotein, which are strong prognostic factors in stroke patients. The interaction between pioglitazone and the risk factors needs to be further investigated. Although we were able to collect entire prescription records, there could have been gaps between prescriptions and actual patient intake of medications. Fortunately, prior studies showed a good correlation between prescription records and actual intake of medications [54, 55]. The determination of outcomes was based on diagnostic codes in the health claims records. Although our method of determination of cardiovascular outcomes based on ICD-10 codes in the NHIS was validated and widely used, records of patients who did not visit the hospital or records containing entry errors may not have been captured [10].

## Conclusions

This study based on nationwide real-world data demonstrated the significant cardiovascular preventive effect of pioglitazone in diabetic patients with acute ischemic stroke. Pioglitazone is currently an available antidiabetic drug, and treatment with pioglitazone may be a promising strategy for reducing residual cardiovascular risk in patients with ischemic stroke and DM who are considered to be high-risk.

## Data Availability

The dataset (NHIS-NSC) supporting the conclusions of this article is available in the homepage of National Health Insurance Sharing Service http://nhiss.nhis.or.kr/bd/ab/bdaba021eng.do. To gain access to the data, a completed application form, a research proposal and the applicant’s approval document from the institutional review board should be submitted to and reviewed by the inquiry committee of research support in NHIS. Currently, use of NHIS data is allowed only for Korean researchers.

## Abbreviations

CI: confidence interval
DM: diabetes mellitus
HR: hazard ratio
ICD-10: International Statistical Classification of Diseases and Related Health Problems 10th Revision
IRIS: Insulin Resistance Intervention After Stroke
J-SPIRIT: Jauntendo Stroke Prevention study in Insulin Resistance and Impaired glucose Tolerance
MI: myocardial infarction
NHIS-NSC: National Health Insurance Service-National Sample Cohort
OR: odds ration
PPAR: peroxisome proliferator-activated receptor
PROactive: PROspective pioglitAzone Clinical Trial In macroVascular Events
TIA: transient ischemic attack
